# National Intensive Care Unit Utilization for Diabetic Ketoacidosis Increased From 2012 to 2022

**DOI:** 10.1111/acem.70099

**Published:** 2025-07-21

**Authors:** Nathan L. Haas, Dan Viderman, Rodney Ahdoot, Kristian Seiler, Richard T. Griffey, Ryan Schneider, James A. Cranford, Alexander T. Janke

**Affiliations:** ^1^ University of Michigan Ann Arbor Michigan USA; ^2^ Washington University in St. Louis St. Louis Missouri USA

The prevalence of diabetic ketoacidosis (DKA) in the United States increased 13.5% annually between 2009 and 2015, and the increasing prevalence of diabetes complications and associated costs pose threat to overwhelm the United States healthcare system, described as the “50‐foot tsunami, 50 yards from shore.” [1, 2] DKA patients are often admitted from the Emergency Department (ED) to the intensive care unit (ICU) with substantial resource utilization and cost. Wide variation in ICU utilization by hospital for DKA exists, ranging from 2.1% to 87.7% [3]. However, ICU capacity is under increasing strain nationally, and as a result the National Academies of Medicine called for strategies to address the increasing mismatch in supply and demand for ICUs to mitigate ICU capacity strain [4]. Pairing the low mortality rate with nationally strained ICU capacity, some consider DKA an “inappropriate” use of ICU resources as DKA competes with other critical conditions in need of limited ICU beds [5]. Strategies to mitigate this by safely managing DKA outside of the ICU have emerged [6, 7]. While the national prevalence of DKA has been quantified [1], there is a paucity of data around annual national ICU utilization for DKA. Our objective was to provide an estimate and describe temporal trends, to better understand opportunities in this space.

We extracted data from the National Hospital Ambulatory Medical Care Survey (NHAMCS), a dataset from a nationally representative sample of hospitals and EDs. We extracted records spanning 2012–2022 (the final year of available NHAMCS data) of ED visits of adults (> 18 years) with a diagnosis of DKA, identified using International Classification of Diseases codes and subcodes: 249.1 and 250.1; E08.1, E09.1, E10.1, E11.1, and E13.1 for 9th and 10th Revisions respectively. The primary outcome was annual ED to ICU admissions for DKA. Additional outcomes include ED visits, hospitalizations, ED discharges, ICU admissions, and respective rates (with ED visits for DKA as denominator). Analyses incorporated sampling weight, cluster, and strata variables to account for complex survey design, and allow for calculation of nationally representative estimates. Domain analyses were utilized to preserve integrity of survey design. Analyses were conducted using SAS software (version 9.4).

From 2012 to 2022, 1,531,254,405 ED visits (95% CI: 1,398,744,514 to 1,663,764,296; 222,175 records), of which 1,935,488 (95% CI: 1,618,554 to 2,252,421; 279 records; 0.13%) were > 18 years with a diagnosis of DKA, were analyzed (Supplement contains demographics). ICU admissions for DKA increased with mean annual percentage change of 17.4% (Figure 1). Annual ED visits, hospitalizations, and ED discharges for DKA increased with average annual percentage changes of 18.6%, 21.0%, and 120.4%, respectively. Among visits with DKA, annual ICU admission rate (mean 39.9%, slope 0.03%), hospitalization rate (mean 81.0%, slope 0.51%), and ED discharge rate (mean 15.5%, slope − 0.76%) remained unchanged. Of ICU admissions with DKA, median hospital length of stay (LOS) was 3.22 days (IQR: 1.76–5.00), and 177,696 (23.4%) had LOS < 48 h.

**FIGURE 1:**
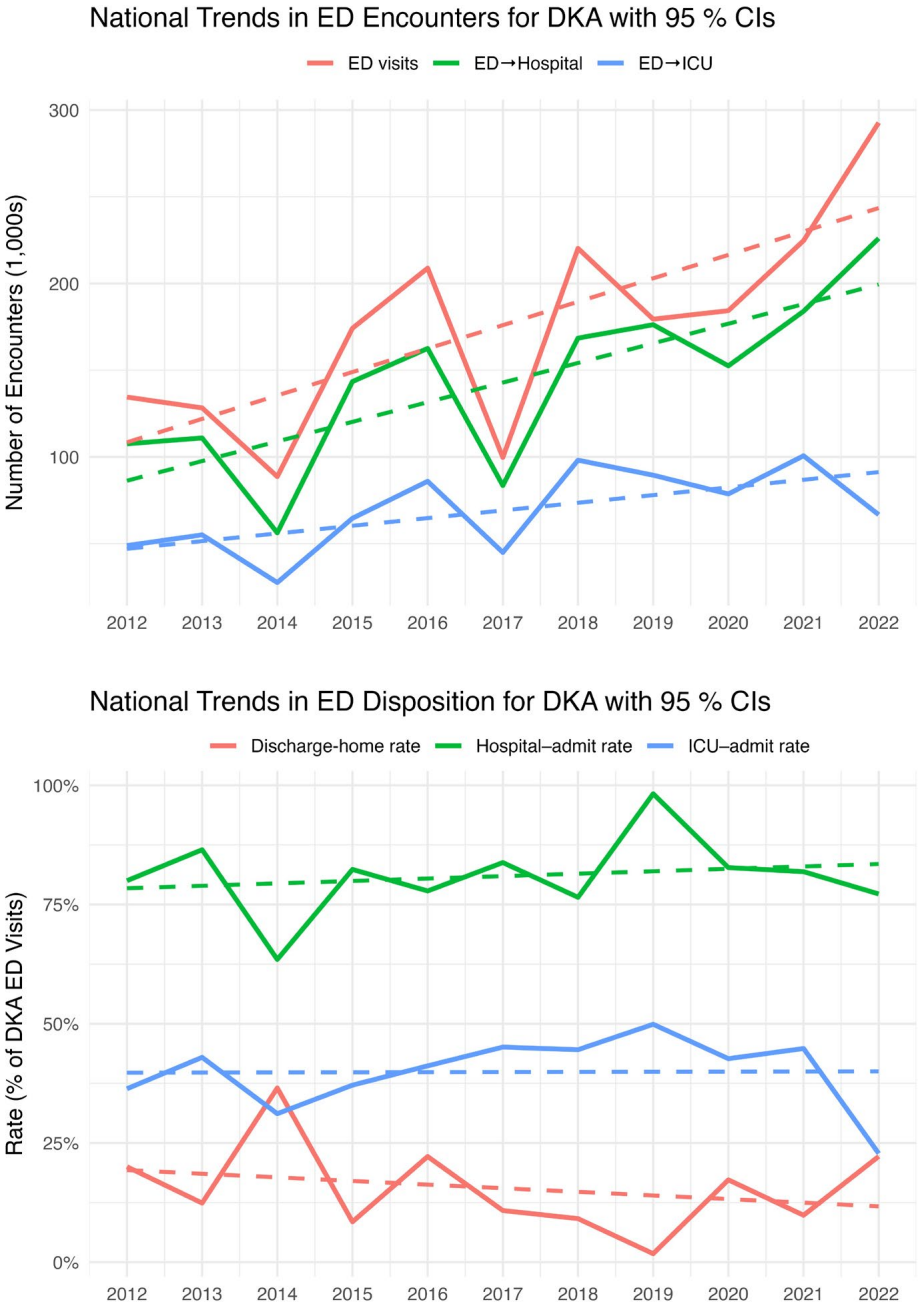
National trends in emergency department encounters and disposition for diabetic ketoacidosis.

ED visits, hospitalizations, and ED to ICU admissions for adults with DKA increased nationally from 2012 to 2022. These data are congruent with reports of increasing DKA prevalence, and provide novel quantification of increasing national ICU utilization for adults with DKA, observing increasing ICU admissions for DKA from 2012 to 2022 with mean annual percentage change of 17.4%. With ICU overcrowding, mitigation strategies to both prevent DKA and safely manage DKA in alternative levels of care are needed.

Comparatively, ED disposition decisions (ICU admission rate, hospitalization rate, and ED discharge rate) remained unchanged. We hypothesize this is due to local practice patterns and policies, more than disease severity or benefits of ICU admission. Significant opportunity exists to prevent ICU admissions for DKA by providing safe strategies targeting local practice patterns, and strategies to do so are emerging [6, 7]. Though the NHAMCS data source does not include stratification by severity, we hypothesize this stability in disposition rates also reflects an unchanged distribution of DKA severity. Thus we hypothesize that, as has been demonstrated previously [6, 8], significant opportunity exists to safely avoid ICU admission for many cases of mild to moderate DKA.

In this study, annual ICU admissions for DKA averaged 64,917. One study reports a cost savings of $10,321 per admission by managing DKA in a step‐down unit rather than ICU. While not all DKA admissions can be managed outside of the ICU (e.g., need for vasopressors or mechanical ventilation), applying these savings to this cohort would save over $1/2 billion annually. Over 20% of national ICU admissions for DKA were “short‐stay ICU admissions” with LOS < 48 h. Some have hypothesized that managing short‐stay ICU admissions in alternative care settings (e.g., step‐down unit or ED‐ICU) may be more cost‐effective [9], and that minimizing short‐stay ICU admissions can optimize limited ICU bed allocation by providing capacity for patients who are decompensating in the wards or by increasing transfers from ICUs in other hospitals [10]. Developing safe strategies for managing DKA in non‐ICU levels of care can contribute to reductions in short‐stay ICU admissions and improvements in resource utilization.

This is a retrospective observational study using a large data set of abstracted information. Limitations include potential for missing data, coding errors, and cross‐sectional data collection. Length of stay data were unavailable for 2016–2017.

In conclusion, ICU admissions for DKA in the United States increased by 17.4% annually from 2012 to 2022, but the proportion of ED patients with DKA admitted to the ICU has remained unchanged. Strategies to safely avoid ICU admission for this increasingly prevalent condition are direly needed to improve limited critical care resource utilization and reduce costs.

## Conflicts of Interest

The authors declare no conflicts of interest.

## Supporting information


Data S1.

## Data Availability

The data that support the findings of this study are available from the corresponding author upon reasonable request.
