# Supplementary material for: National Intensive Care Unit Utilization for Diabetic Ketoacidosis Increased From 2012 to 2022
Source: Acad Emerg Med. 2025 Jul 21;32(11):1248–50. doi: 10.1111/acem.70099 (PMC12611341; doi:10.1111/acem.70099)
Supplement: Supplementary file 1 — Data S1. [file ACEM-32-1248-s001.docx]

**Supplement**:

Demographics of ED visits of adults with DKA

Age

<25 years: 288,537 (14.91%)

25-49 years: 947,556 (50.35%)

≥50 years: 672,394 (34.74%)

Sex

Female: 943,833 (48.30%)

Male: 1,000,655 (51.70%)

Race

White: 1,370,585 (70.81%)

Black: 504,783 (26.08%)

Other race: 60,119 (3.11%)

Primary payor

Blank primary payor: 10,321 (0.53%)

Unknown: 119,227 (6.16%)

Private insurance: 505,652 (26.13%)

Medicare: 400,623 (20.70%)

Medicaid or CHIP or other state-based program: 615,246 (31.79%)

Worker’s compensation: 1,438 (0.07%)

Self-pay: 190,865 (9.86%)

No charge / charity: 3,816 (0.20%) no charge / charity

Other primary payor: 88,300 (4.56%)

Day of ED visit

Monday-Friday: 1,541,596 (79.65%)

Saturday-Sunday: 393,891 (20.35%)
